# Maternal and Fetal Metabolites in Gestational Diabetes Mellitus: A Narrative Review

**DOI:** 10.3390/metabo12050383

**Published:** 2022-04-22

**Authors:** Ionela Mihaela Vladu, Diana Clenciu, Adina Mitrea, Anca Amzolini, Simona Elena Micu, Anda Elena Crisan, Ion Cristian Efrem, Maria Fortofoiu, Mircea Catalin Fortofoiu, Adrian Mita, Anca Barau Alhija, Adina Dorina Glodeanu, Maria Mota

**Affiliations:** 1 Department of Diabetes, Nutrition and Metabolic Diseases, Faculty of Medicine, University of Medicine and Pharmacy of Craiova, 200349 Craiova, Romania; ionela.vladu@umfcv.ro (I.M.V.); dianaclenciu@yahoo.com (D.C.); mmota53@yahoo.com (M.M.); 2 Department of Internal Medicine, Medical Semiology, Faculty of Medicine, University of Medicine and Pharmacy of Craiova, 200349 Craiova, Romania; anca.amzolini@umfcv.ro (A.A.); simona.micu@umfcv.ro (S.E.M.); catalin.fortofoiu@umfcv.ro (M.C.F.); adrian.mita@umfcv.ro (A.M.); anca.barau@umfcv.ro (A.B.A.); 3 Department of Oncology, Faculty of Medicine, University of Medicine and Pharmacy of Craiova, 200349 Craiova, Romania; anda.crisan@umfcv.ro; 4 Department of Internal Medicine and Medical Semiology, Faculty of Dentistry, University of Medicine and Pharmacy of Craiova, 200349 Craiova, Romania; cristian.efrem@umfcv.ro (I.C.E.); adina.glodeanu@umfcv.ro (A.D.G.); 5 Department of Emergency Medicine, Faculty of Medicine, University of Medicine and Pharmacy of Craiova, 200349 Craiova, Romania

**Keywords:** gestational diabetes mellitus, metabolomics, obesity, cardiovascular risk, biomarkers, metabolic pathways

## Abstract

Gestational diabetes mellitus (GDM) is a major public health issue of our century due to its increasing prevalence, affecting 5% to 20% of all pregnancies. The pathogenesis of GDM has not been completely elucidated to date. Increasing evidence suggests the association of environmental factors with genetic and epigenetic factors in the development of GDM. So far, several metabolomics studies have investigated metabolic disruptions associated with GDM. The aim of this review is to highlight the usefulness of maternal metabolites as diagnosis markers of GDM as well as the importance of both maternal and fetal metabolites as prognosis biomarkers for GDM and GDM’s transition to type 2 diabetes mellitus T2DM.

## 1. Introduction

Hyperglycemia in pregnancy is associated with a higher risk of adverse outcomes, such as spontaneous abortion, pre-eclampsia, fetal anomalies, fetal macrosomia, shoulder dystocia, neonatal hypoglycemia, neonatal respiratory distress syndrome, and even perinatal death [1], as well as increased cardiovascular risk for both mother and offspring. According to the World Health Organization (WHO) and the International Federation of Gynaecology and Obstetrics (FIGO), hyperglycemia in pregnancy includes pre-gestational diabetes mellitus, overt diabetes mellitus diagnosed in pregnancy, and gestational diabetes mellitus (GDM) [2,3,4]. The International Diabetes Federation (IDF) estimated that in females aged 20–49 years old, hyperglycemia in pregnancy affected 16.7% of all live births, corresponding to 21.1 million live births, in 2021, the majority of the cases presenting with GDM (80.3%) [2].

For many years, GDM was defined as previously unknown glucose intolerance diagnosed during pregnancy. As this definition has important limitations, it was revisited and currently the accepted definition of GDM is diabetes diagnosed during pregnancy, but only in the second and third trimesters [5,6]. The American Diabetes Association (ADA) defines GDM after performing an oral glucose tolerance test (OGTT) during 24–28 weeks of gestation, using either a one-step strategy (International Association of Diabetes and Pregnancy Study Groups criteria) or a two-step strategy (Carpenter and Coustan’s criteria) [5]. However, since 1964, when GDM was first defined [7], there are multiple criteria still used around the world, complicating the interpretation of different epidemiological studies related to GDM prevalence, which was estimated to 5–20% of all pregnancies [8,9,10].

It is universally accepted that females diagnosed with GDM are at an increased risk for the development of type 2 diabetes mellitus (T2DM). Furthermore, a number of studies have suggested that patients with GDM also have a higher cardiovascular risk, a recent meta-analysis proving that these females have a twofold higher risk of cardiovascular events, not dependent upon the intercurrent T2DM development [11]. Therefore, we consider that T2DM and cardiovascular disease prevention are essential in females with a history of GDM, as T2DM is associated with heart failure, chronic macrovascular complications (atherosclerosis and ischemic events) [12], and microvascular complications [13], such as chronic kidney disease, which in a recent study was described as a marker of heart failure severity [14]. T2DM is also associated with alterations in the lipid profile [15], a disorder that in the presence of other uncontrolled cardiovascular risk factors, such as uncontrolled hypertension, can put the patient at risk for a variety of severe disorders, ranging from vascular events to abdominal aorta aneurysm [16].

Molecules having a molecular weight <1000 Da (metabolites) resulting from all metabolic pathways are analyzed qualitatively or quantitatively using metabolomics studies [17]. Nicholson et al. described for the first time this scientific study [17,18]. Metabolites grant a glance into the biological processes through the exhibition of molecular by-products [19]. Metabolomics provides the end points of different biological processes under the influence of nutritional, environmental, and pharmaceutical factors, giving sensitive results regarding metabolites from distinctive cells, tissues, and biological fluids [19]. Furthermore, through metabolomics, information related to the molecular contents of the organism as a whole is also supplied [19]. There are thousands of known and unknown human metabolites, with important variations regarding size, polarity, and concentration, the detection, identification, as well as the quantifications of these molecules being challenging and requiring special techniques, such as mass spectrometry (MS) and nuclear magnetic resonance (NMR) spectroscopy [17]. Metabolomics has been recently acknowledged as a helpful tool for the identification of metabolic disruptions associated with cardiovascular and metabolic diseases, including T2DM and GDM [20].

Metabolomics studies are trying to elucidate the mechanisms of atherosclerosis and cardiovascular, metabolic, and other noncommunicable diseases [21]. Most cardiovascular diseases are associated with disturbances of cardiac metabolism, as this organ requires a constant energy supply [22]. The introduction of metabolomics into epidemiologic studies is useful for the understanding of gene–environment interaction consequences, as well as for the discovery of novel biomarkers helpful for the early prevention and detection of atherosclerosis and cardiovascular diseases [21,22]. What is more, metabolism disturbances observed in metabolic diseases, such as obesity and diabetes, have a direct impact upon heart metabolism, which, in turn, will further impair systemic metabolism [22]. Therefore, metabolomics studies can bring to light disturbances in both cardiac and systemic metabolisms that may lead to the vicious circle responsible for the initiation and perpetuation of cardiovascular diseases [22]. The potential importance of metabolomics in cardiovascular health was also recognized by the American Heart Association in 2017 in a statement that debated the current challenges of metabolomics studies and their possible clinical applications [23]. Furthermore, metabolites such as select unsaturated lipid species, trimethylamine-*N*-oxide, and branched-chain amino acids are recognized as biomarkers of cardiovascular diseases [23].

The role of metabolomics studies in recognizing potential early biomarkers of GDM is even more important taking into account that at the moment there are no early diagnosis criteria for this disorder. In the last two decades, several narrative or systematic reviews concerning metabolomics studies in GDM have been published in the medical literature. Among these studies, we consider worth mentioning the paper published by Huynh et al. in 2014 [24]. In this review, the authors evaluated the existing literature at that moment in a rigorous and detailed way, concluding that the analyzed studies presented inconsistent results, and highlighted the need for larger studies that could bridge the gap between clinical practice and clinical research by conducting metabolomics studies on samples obtained early during pregnancy [24]. Since 2014, many other review papers have tried to bring forward the usefulness of metabolomics studies in understanding the disrupted metabolic pathways associated with GDM [8,17,20,25,26,27,28]. 

The aim of our review is to highlight the metabolic disruptions associated with GDM by metabolomics studies, supplementing data presented in previous papers with the results of recently published studies. Furthermore, our paper complements previous reviews by also including fetal metabolomics studies. Through this narrative review, we do not intend to bring forward new conclusions regarding metabolomics studies in GDM. Our intention is to outline the usefulness of some maternal metabolites as early diagnosis markers of GDM as well as the importance of both maternal and fetal metabolites as prognosis biomarkers for GDM and GDM’s transition to T2DM. Moreover, understanding the results of relevant metabolomics studies in GDM can facilitate the early diagnosis and treatment of this disorder, preventing adverse effects in both mothers and babies.

## 2. Maternal Metabolites in Gestational Diabetes Mellitus

In the last few decades, many metabolomics studies have tried to explain the role of different maternal metabolites in the development of GDM, analyzing a variety of biological samples, ranging from plasma and urine, which are used in the majority of the studies, to erythrocyte membrane, amniotic fluid, placenta, breast milk, and even hair [14]. Furthermore, maternal metabolite studies differ also in regard to sample collection time (first, second, or third trimester of pregnancy; at delivery; or postpartum) as well as in terms of the studied intermediate metabolism (carbohydrates; lipid metabolites, such as phospholipids, sphingomyelin, and fatty acids; amino acids and purines; uric acid; bile acid, etc.) [14,17]. Table 1 summarizes selected metabolite studies in GDM.

### 2.1. Maternal Carbohydrate Metabolites in Gestational Diabetes Mellitus

The main characteristic of GDM is the increased blood glucose level, which keeps increasing in the second and third trimesters of pregnancy, justifying the need for metabolomics studies for the identification of carbohydrate metabolites with implications in GDM as well as in the transition from GDM to T2DM.

GDM is recognized as a complex and multidimensional disorder. Metabolomics studies proved the enhanced gluconeogenesis early in the first pregnancy trimester, prior to the diagnosis of incident dysregulation of blood glucose, from substrates such as alanine, glutamic acid, and pyruvic acid [33].

Mokkala et al. performed a metabolomics study in females who were overweight or obese, the primary outcome being the differences met in serum metabolic profile in late pregnancy in females diagnosed with GDM vs. women without GDM [29]. The authors reported that females with GDM have higher levels of citrate, recognized as a regulator of energy metabolism, through glycolysis inhibition and gluconeogenesis stimulation [29,43]. In addition to these findings, the study performed by Gralka et al. in non-pregnant females demonstrated that citrate and pyruvate may be determinants of adiposity in females who were overweight or obese [44].

Recent studies have evidenced increased levels of hexose (defined as the sum of glucose, fructose, and other hexoses) both in females with GDM and in females with GDM who over a period of 2 years postpartum developed T2DM [30,32]. The study performed by Shokry et al. showed that the sum of hexoses measured at delivery was significantly increased in females with GDM [32]. Lai et al. showed a highly significant association between hexose and future T2D risk at baseline as well as increased hexose levels over the study observation period in the patients that developed T2DM compared to those who remained normoglycemic [30]. Therefore, the sum of hexoses is a biomarker relevant to the relationship between GDM and T2DM. Further studies are necessary in order to identify carbohydrate metabolites that could be used as early GDM biomarkers.

### 2.2. Maternal Lipid Metabolites in Gestational Diabetes Mellitus

Lipid molecules play roles in many important life processes, such as energy conversion, information recognition, and material transport, in addition to development and differentiation of cells, as well as apoptosis [17]. Therefore, it is understandable that an impaired lipid metabolism is associated with many disorders, including obesity, diabetes, and GDM, in the latter scenario, lipids’ physiological changes being magnified, suggesting metabolic impairments during pregnancy [17,45]. 

Pregnancy is associated with both physiological insulin resistance and lipid metabolism alterations, which are more evident starting 24–28 weeks of gestation [46,47]. In normal pregnancy, however, although there is an increase in total cholesterol, triglycerides, and HDL-cholesterol, the atherogenic index (LDL-cholesterol to HDL-cholesterol ratio) remains unchanged [46]. In the case of GDM, the changes in lipid profile and insulin resistance are exacerbated, with a shift toward greater small, dense LDL subtractions, typical for insulin resistance, proving an underlying transient metabolic dysfunction [46,47]. Regarding altered lipid metabolites in GDM, studies have described different lipid particles (fatty acids, glycerolipids, phospholipids, glycerophospholipids, and sphingolipids) that are associated with this disorder.

Saturated fatty acids and monounsaturated fatty acids stand out from the fatty acid family as they are involved in maintaining cell membrane fluidity, in cell proliferation, and in programmed cell death, being linked to insulin resistance, obesity, diabetes, and other metabolic diseases and cancers [17,48,49,50,51]. It is believed that different fatty acids could participate in maintaining the health of both mother and offspring. Furthermore, it is possible that the same fatty acid can act differently in relation to the exposure times and its dose. In a recent study, Odenkirk et al. analyzed fatty acyl groups in patients with GDM and demonstrated a downregulation in lipids containing 12:0, 14:0, 15:0, 18:3, 22:4, or 24:1 fatty acyls, as well as an upregulation in lipids containing 20:0 and 22:6 fatty acyls [36]. Contrary to previous studies [17], myristic acid (previously associated with 22:6) was also downregulated, although not in the same species of lipids [36]. Another study, including 107 females diagnosed with GDM and 107 matched healthy controls, performed by Zaho et al. showed that palmitoyl carnitine and vaccenyl carnitine, long-chain acylcarnitines, were increased in the serum of females with GDM, both in the first and second trimesters of pregnancy, making these metabolites possible early GDM biomarkers [52]. Raczkowska et al. proposed a different approach, studying lipid metabolites after classifying the glucose impairments found in GDM as abnormal fasting plasma glucose—GDM (aFPG-GDM) and abnormal glucose tolerance—GDM (aGT-GDM) [34]. Differences related to capric, caprylic, heptadecanoic, lauric, myristic, nonanoic, oleic, palmitic, palmitoleic, and stearic acids, as well as α-hydroxybutyric and β-hydroxybutyric acids, were observed [34]. What is more, this study proposed a combination of three lipid metabolites (myristic acid and both hydroxybutyric acids) that were strongly associated with aGT-GDM as potential biomarkers of GDM in the first and second trimesters of pregnancy [34]. The authors supported their biomarkers on the high specificity and sensitivity of this combination, having an area under curve (AUC) of 0.828 for aGT-DM [34]. Furthermore, the combination of the three biomarkers is also highly sensitive and specific in selecting females at risk for aGT-DM in the first trimester of pregnancy, with an AUC of 0.791 [34]. Other case-control studies also proved that β-hydroxybutyric acid had a higher concentration in patients with GDM [29,53,54], findings that were also reported by Mokkala et al. [29]. It is believed that the appearance of ketone bodies in GDM is due to the failure of glucose utilization, similar to the processes met in patients with T2DM. Furthermore, as pregnancy progresses, maternal metabolism is dominated by catabolic processes, increased lipolysis providing fatty acids as substrates for ketogenesis, which are used as maternal energy as the fetus uses mainly glucose [17,55,56].

Furthermore, studies have taken into account that the fatty acid profile of breast milk might be related to developmental disorders and even health risk for babies born to mothers with GDM. Such a study conducted by Wu et al. [57] assessed the metabolic profile of breast milk samples (colostrum, transition milk, and mature milk) from females with GDM and females with normal glucose tolerance. This study proved a modified breast milk lipid profile in females with GDM, with an increase in two unsaturated lipids, eicosatrienoic acid and lysophosphatidylcholine, both of them being associated with excessive neonatal body weight gain [57]. 

Regarding the roles of sphingomyelin and phospholipids in diabetes and GDM pathogenesis, the studies are still scarce and have reported inconsistent results. A study focused on lipid metabolite alteration in GDM as well as the lipid metabolite profile in females that transitioned from GDM to T2DM evidenced that whilst 11 acyl-alkyl-glycerolphospholipids were negatively correlated with the progression to T2DM, six diacyl-glycerolphospholipids were associated with this transition, presenting elevated levels both at baseline (postpartum) and at the follow-up visit (after 2 years) [30]. The same study reported the lack of association between some sphingomyelins and the risk of T2DM [30], confirming the results of Floegel et al., who detected that some diacyl-phosphatidylcholines were related with an increased risk of T2DM, albeit sphingomyelins and 1 acyl-alkyl-phosphatidylcholine presented a lower risk [58]. What is more, disturbances in the metabolism of particular classes of phospholipids and lysophospholipids were observed before hyperglycemia at 2 years postpartum (in the group at risk for diabetes) in a hyperglycemia and pregnancy adverse outcome study [59]. The results of these studies suggest that goglycerophospholipid metabolism is involved in the transition to T2DM. However, when interpreting the results reported by Lai et al. [30], we must also take into account conflicting evidence, supporting the role of sphingomyelin metabolism in T2DM, in addition to its association with increased risk of developing T2DM, presented by other studies [60,61], making necessary the further study of sphingomyelin metabolism in GDM and T2DM.

The altered lipid metabolites in females with GDM were also described by Zhan et al. in a recent case-control study [31]. The study identified a significant increase in metabolites from glycerophospholipid metabolism (phosphatidylethanolamine, phosphatidylcholine, phosphatidylinositol, lysophosphatidylcholine, lysophosphatidylethanolamine, lysophosphatidic acid, cyclic phosphatidic acid, and 1-lyso-2-arachidonoyl-phosphatidate), as well as altered levels of 17-Hydroxydocosahexaenoic acid, cortisol, and carnitine metabolites [31]. Furthermore, the authors pinpointed five lipid metabolites, phosphatidylcholine (22:6(4Z,7Z,10Z,13Z,16Z,19Z)/P-18:1(11Z)) with a fold change of 0.601, phosphatidylethanolamine (22:2(13Z,16Z)/P-18:1(11Z)) with a fold change of 0.4332, monoacylglycerol (15:0/0:0/0:0) with a fold change of 0.6629, lysophosphatidylethanolamine (0:0/18:0) with a fold change of 0.3848, and cyclic phosphatidic acid (16:0/0:0) with a fold change of 0.3466, that were altered in both second and third pregnancy trimesters [31]. The receiver operating characteristic curve (ROC) analysis was performed in order to evaluate whether these five metabolites can be used for the diagnosis of GDM, evidencing AUCs between 0.701 and 0.873 in the second trimester and values between 0.702 and 0.889 in the third trimester, thus demonstrating the high diagnostic performance of these biomarkers [31]. Based on a pathway and pathway topology analysis, the authors concluded that the most affected pathway in GDM was glycerophospholipid metabolism [31].

### 2.3. Maternal Amino Acid Metabolites in Gestational Diabetes Mellitus

An association of amino acid metabolites, including aromatic amino acids and branched-chain amino acids, with the risk of obesity, insulin resistance, and T2DM was recognized more than 40 years ago [62]. Amino acids are a part of many metabolic pathways related to insulin resistance, such as the mammalian target of rapamycin (mTOR), Insulin receptor substrate 1 (IRS1), c-Jun N-terminal kinase (JNK), and fatty acid oxidation, as well as hyperinsulinemia associated with pancreatic β-cells exhaustion, as amino acid metabolites being involved in insulin secretion modulation [62,63,64]. Taking into consideration these aspects, a question was raised related to the roles played by amino acid metabolites in the metabolic disturbances linked to GDM. Early pregnancy was associated with abnormal amino acid metabolism, leading to the development of GDM; however, no consensus exists regarding the utility of amino acids as early biomarkers of GDM [65]. A study analyzing these issues [29] revealed that isoleucine and leucine (branched-chain amino acids) as well as alanine and phenylalanine were increased in the first pregnancy trimester (<18 weeks of gestation) in females that later were diagnosed with GDM, confirming previous results regarding elevated amino acids (valine and phenylalanine, but not alanine) in females with obesity and GDM [66]. Increased levels of glutamate, alanine, phenylalaine, tyrosine, and isoleucine in early pregnancy in females with GDM were also reported by Jiang et al. [40]. Other studies that have enrolled females with heterogeneous body mass indexes have demonstrated that the onset of GDM was associated with increased levels of alanine [17,67,68] and valine [67], but without any changes in branched-chain amino acids [68,69], in early pregnancy [17]. Other amino acid metabolites that have been proposed as being predictive for GDM in early pregnancy include serotonin, melatonin, 5-hydroxy-indol-acetic acid, L-tryptophan, and 6-hydroxymelatonin [70]. Furthermore, in a study conducted by Sakurai et al., in early pregnancy, glutamine in serum and ethanolamine in urine showed AUCs > 0.80 being candidate early predictive biomarkers for GDM [41].

In the study conducted by Zhan, an increase in lysyl-tyrosine (a dipeptide resulting from protein degradation [71]) was associated for the first time with GDM, having a fold change of 0.4673 [31]. In the light of all these metabolomics studies, it is understandable why the inclusion of amino acid measurement was suggested as routine testing in early pregnancy in order to identify females at risk of developing GDM [40,72,73,74].

Andersson-Hall et al. demonstrated that the disturbances in amino acid metabolism associated with GDM are also predictive for the progression to T2DM [75]. These conclusions confirm previous results published by Wheeler’s group [76]. This study proved that leucine, isoleucine, tyrosine, tryptophan, amino phenylalanine, and alanine were predictors for the development of T2DM within 2 to 4 years after delivery in females diagnosed with GDM [76]. Furthermore, Floegel et al. showed that the transition to T2DM is related to the enhancement of insulin resistance due to the high plasma levels of hydroxyisobutyric acid and branched-chain amino acids [58]. Several other animal and human studies, however, did not find significant differences between females with GDM and the normal glucose tolerance control group regarding the concentrations of branched-chain amino acids throughout pregnancy and could not prove unequivocally the usefulness of restricting or supplementing branched-chain amino acids in order to modify T2DM risk development [68,77,78,79,80].

In this light, we consider that further metabolomics studies related to amino acid metabolites in GDM are still needed in order to draw pertinent conclusions and to explore the molecular mechanisms linking these metabolites to GDM and T2DM.

### 2.4. Other Maternal Metabolites in Gestational Diabetes Mellitus

Studies have also concentrated on highlighting immune dysfunctions associated with pregnancy complications in females with GDM. In this regard, glycoprotein acetylation and selected steroid hormones (cortisol, 11-deoxycortisol, progesterone, 17α-hydroxyprogesterone, androstenedione, and dehydroepiandrosterone sulfate), considered low-grade inflammatory markers, were associated with GDM [29,66,67,81,82]. 

Bile acids are other metabolites linked to GDM by metabolomics studies. Two recent studies performed by Yang’s group [83,84] recorded a decrease in deoxycholic acid and glycoursodeoxycholic acid trimethylamine nitrogen oxide in early pregnancy. Moreover, increased trimethylamine was linked to an increased risk of GDM [83,84]. 

The association between purine metabolism and T2DM and GDM is generally recognized, hyperuricemia being a trademark of insulin resistance [85,86,87]. A recent study [52] has demonstrated that hypoxanthine and xanthine, purine metabolites, were significantly higher in females with GDM in early pregnancy. The increased activity of oxidase in GDM can explain why in the second trimester there is a decrease in circulating purine metabolite levels, parallel to the increase in uric acid concentration [17]. Zaho’s study confirmed the results obtained two years previously by Law et al., who in a study of the urine metabolome reported that tryptophan and purine metabolism were linked to the progression of GDM [88]. Furthermore, the authors of this study noted that in patients with GDM, the metabolic pathway involved in the production of nicotinamide adenine dinucleotide from tryptophan was activated before any physiological changes could be induced by the hormones produced by the placenta or by the fetoplacental unit [88]. The findings of this study challenge traditional views related to the pathogenesis of GDM, placing an emphasis on the possibility that GDM is actually a predisposed condition, i.e., a pre-existing metabolically altered pre-diabetic state, that is fully realized during pregnancy [20,88].

## 3. Associations of Gestational Diabetes Mellitus with Fetal Metabolites

Although the last two decades have brought an abundance of metabolomics studies in GDM that have assessed maternal metabolites, studies that are dedicated to association between fetal metabolome and GDM are still scarce. In a study that included 412 pregnant females, Lu et al. aimed to compare fetal metabolites circulating in cord blood of newborns from patients with GDM to those met in children born to mothers with normal glucose tolerance, using flow injection analysis—electrospray ionization—tandem mass spectrometry [89]. After adjusting for different risk factors associated with GDM, the authors of the study identified phosphatidylcholine acyl-alkyl C 32:1 and proline in cord blood to be independently associated with GDM [89]. The medical literature has linked GDM with an increased risk of preterm birth [90]. The study conducted by Lu et al. confirmed this finding and also demonstrated that cord blood PC ae C32:1 and proline concentrations might influence the effect of GDM on gestational age [89]. Regarding the identified metabolites, previous studies have also found a correlation between proline measured both in neonatal blood and amniotic fluid and the risk of premature birth [91,92,93].

An older study that assessed cord blood from children born to mothers with GDM [94] recorded higher circulating levels of amino acids, pyruvate, histidine, alanine, methionine, arginine, lysine, hypoxanthine, lipids, and lipoproteins together with a decreased glucose level. Furthermore, Pang et al. [95] in a meconium metabolome study noted alteration in purine, lipid, and amino acid metabolisms in the offspring of mothers with GDM.

A study published in 2019 assessed metabolites associated with GDM from both maternal serum and cord blood [32], evidencing elevated levels of sum of hexoses in both types of samples. Moreover, the study described metabolites’ particularities only in the cord blood of babies born to mothers with GDM, such as a significantly lower level of free carnitine, acyl carnitines, non-esterified fatty acids, phospholipids, β-oxidation markers, and metabolites of the Krebs cycle [32]. The authors tried to explain these findings by the reduced transport of carnitine and fatty acid oxidation, consequences of fetal hyperglycemia, a process that have also been described in insulin resistance states, such as diabetes [32].

Given the lack of studies that have evaluated fetal metabolome, it is hard to draw a conclusion regarding the association of cord blood or meconium metabolites with the pathogenesis of GDM. Further prospective studies that follow-up both patients with GDM and their babies might evidence metabolic disruptions that put these subjects at greater risk for T2DM and cardiovascular diseases. 

## 4. Conclusions

Metabolomics studies in GDM are concentrated on disturbances in carbohydrate, lipid, and amino acid metabolites, as well as sterol hormones, purine, and bile acid metabolites, emphasizing disordered metabolic pathways related to these metabolites. The results provided by metabolomics studies are of clinical importance, especially when we are discussing metabolites that early in the course of pregnancy (first trimester) are associated with GDM and T2DM risk, facilitating early interventions meant to prevent or diminish adverse outcomes described in GDM. Clinical utility can be attributed also to studies focused on the second and third pregnancy trimesters, as their conclusions put forward important data about altered metabolic pathways, in addition to the prediction of both pregnancy outcomes and maternal–fetal prognosis. The metabolomics studies performed to date have tried to evidence biomarkers associated with GDM, some of these studies proposing early predictors of GDM based on sensitivity and specificity obtained. However, the usefulness of these screening/diagnosis biomarkers is yet to be confirmed, as most of the studies include a small number of patients or lack validation cohorts, future research being required.

Postpartum follow-up metabolomics studies supply important information related to the transition from GDM to T2DM, but at the moment, there is a knowledge gap that needs to be addressed in future studies regarding the impact of GDM history on offspring born to mothers with GDM.

In conclusion, it is expected that metabolomics studies on GDM, which are rapidly progressing, will make an important contribution to both the prevention and treatment of GDM and T2DM. 

## Figures and Tables

**Table 1:** Selected metabolomics studies in gestational diabetes mellitus.

Assessed Metabolism	Ref	Type of Study	Biological Sample	GDM Criteria	Sample Collection Time	Metabolic Platform	Altered Metabolites in GDM
Carbohydrate, lipid, and amino acid metabolism	Mokkala et al., 2020 [29]	Prospective study (100 patients with GDM vs. 252 women without GDM)	Plasma	IADPSG criteria	Before GDM diagnosis (late pregnancy)	NMR	Citrate (intermediate metabolite in the tricarboxylic acid cycle) levels were higher in females with GDM. All-sized VLDL particles, medium-sized HDL particles, and small-sized HDL particles were higher, while large HDL particles were decreased in females with GDM in early pregnancy. VLDL particles remained higher in females with GDM in the third trimester of pregnancy, while small HDL particles were decreased. There were increased levels of isoleucine, leucine, phenylalanine, and alanine in early pregnancy in females that developed GDM.
Carbohydrate and lipid metabolism	Lai et al., 2020 [30]	Prospective cohort study (173 incident T2D cases and 485 non-T2D controls)	Plasma	Carpenter and Coustan criteria	From 6–9 weeks postpartum (baseline) up to 2 years (follow-up)	FIA–MS	There were increased hexose levels in patients with GDM that developed T2DM. Six diacyl-glycerolphospholipids were positively correlated with the transition from GDM to T2D at baseline and follow-up, while 11 acyl-alkyl-glycerolphospholipids were negatively correlated with T2D risk.
Lipid and amino acid metabolism	Zhan et al., 2021 [31]	Case-control study (49 patients with GDM and 54 healthy pregnant women)	Serum	IADPSG criteria	After GDM diagnosis (24–27 or 28–36 weeks of gestation)	UHPLC–QTOFMS	Glycerophospholipids were the most altered compounds in females with GDM. Monoacylglycerol, dihydrobiopterin, and 13S-hydroxyoctadecadienoic acid were identified with strong discriminative power for GDM. There were increased levels of lysine-tyrosine and L-arginine.
Carbohydrate metabolism	Shokry et al., 2019 [32]	Case-control study (45 mothers with GDM; 67 healthy, normal-weight mothers; and 50 healthy, overweight/obese mothers)	Plasma	National Diabetes Data Group criteria	At delivery	LC–MS/MS	Sum of hexoses (about 90–95% glucose and 5% other hexoses) was significantly higher in GDM.
	Hou et al., 2018 [33]	Nested case-control study (131 GDM cases and 138 controls)	Plasma	IADPSG criteria	Before GDM diagnosis (about 12 weeks of gestation)	UPLC–QTOFMS, UPLC–TQMS, GC–TOFMS	Enhanced gluconeogenesis was suggested by increased alanine, glutamic acid, and pyruvic acid.
Lipid metabolism	Raczkowska et al., 2021 [34]	Case-control study (discovery phase: 79 pregnant women (50 women diagnosed with GDM and 29 controls); validation cohort consisted of 163 pregnant women (95 women with GDM and 68 controls))	Serum	IADPSG criteria	Total 92 females: before GDM diagnosis (8–14 weeks of gestation) Total 662 females: at GDM diagnosis (24–28 weeks of gestation)	GC–MS	A combination of α–hydroxybutyric acid, β–hydroxybutyric acid, and myristic acid was found to be highly specific and sensitive for the diagnosis of GDM manifested by altered glucose tolerance (i.e., GDM) or to select women at a risk of altered glucose tolerance (i.e., GDM in the first trimester).
	Liu et al., 2020 [35]	1:1 Nested case-control study (486 pregnant females)	Plasma	IADPSG criteria	Before GDM diagnosis (9–11 weeks of gestation)	LC–MS/MS	Lysophosphatidylcholines (LPC, e.g., LPC15:0, LPC17:0, LPC18:0, and LPC18:1) were higher in females with GDM.
	Odenkirk et al., 2020 [36]	Case-control study (45 women with GDM, 48 women with term pre-eclampsia, and 98 healthy control women)	Plasma	IADPSG criteria	At the time of labor and delivery (at the time of admission, after admission, and up to 24 h after delivery)	LC–IMS–MS	Lipids containing 12:0, 14:0 (including myristic acid), 15:0, 18:3, 22:4, or 24:1 fatty acyls were downregulated in females with GDM, while 20:0 and 22:6 fatty acyls were upregulated.
	Liu et al., 2019 [37]	Case-control study (23 women with GDM and 22 females without GDM)	Serum	IADPSG criteria	27–33 weeks of gestation	GC–MS/MS	Lysophosphatidylcholines, sphingomyelins, and ceramides were significantly increased in females with GDM and hyperlipidemia.
	Li et al., 2019 [38]	Case-control study (30 GDM patients and 30 healthy pregnant women)	Serum	IADPSG criteria	GDM diagnosis (24–28 weeks of gestation)	LC–MS	Females with GDM presented increased levels of traumatic acid, pravastatin, 2S-hydroxybutanoic acid, D(-)-beta-hydroxy butyric acid, 4-hydroxy-butyric acid, oleic acid, rumenic acid, linoleic acid, corticosterone, 11-deoxycortisol, tetrahydrocortisol, 2-hydroxyestrone, dehydroepiandrosterone sulfate, and tetrahydrocorticosterone.
	Rahman et al., 2018 [39]	Prospective study (107 GDM and 214 non-GDM women)	Plasma	Carpenter and Coustan criteria	Before GDM diagnosis (8–13 and 16–22 weeks of gestation), at diagnosis, and after diagnosis of GDM (24–29 and 34–37 weeks of gestation)	GC–MS	Mid-to-long carbon chain glycerolipids were associated with GDM.
Amino acid metabolism	Jiang et al., 2020 [40]	Case-control study (431 women, of whom 65 developed GDM)	Serum	IADPSG criteria	Before GDM diagnosis (<12–16 weeks of gestation)	UHPLCMS/MS	Glutamate, alanine, phenylalaine, tyrosine, and isoleucine were increased in GDM.
	Sakurai et al., 2019 [41]	Case-control study (121 GDM and 121 non-GDM women)	Plasma and urine	Two-step strategy	Before GDM diagnosis (<16–19 weeks of gestation)	HILICMS/MS	In serum, glutamine, pyrophosphate, and octulose-1,8-bisphosphate significantly differed between females with DM and the ones with normal glucose tolerance. In urine, significant differences were found for shikimate-3-phosphate, ethanolamine, 1,3-diphosphoglycerate, and N-acetyl-L-alanine.
	O’Neill, et al., 2018 [42]	Nested case-control study (20 females with GDM)	Amniotic fluid	Females with GDM confirmed prior to the study enrolment	Second trimester	GC–MS	Glycine, lysine, glutamine, histidine, tryptophan, phenylalanine, and arginine were altered in the amniotic fluid samples from females with GDM.

FIA–MS: flow injection analysis–tandem mass spectrometry; GC–MS: gas chromatography–mass spectrometry; GCMS/MS: Gas chromatography–tandem mass spectrometry; GC–TOFMS: gas chromatography–time-of-flight mass spectrometry; HILIC–MS/MS: hydrophilic interaction chromatography–tandem mass spectrometry; LC–IMS–MS: liquid chromatography–ion mobility spectrometry and mass spectrometry; LC–MS: liquid chromatography–mass spectrometry; LC–MS/MS: liquid chromatography–tandem mass spectrometry; NMR: proton nuclear magnetic resonance; UHPLC–MS/MS: ultra-high-performance liquid chromatography–tandem mass spectrometry; UPLC–QTOFMS: ultra-performance liquid chromatography–quadrupole-time of flight-mass spectrometry; UPLC–TQMS: ultraperformance liquid chromatography–triple-triple-quadrupole-mass spectrometry.

## Data Availability

Not applicable.
